# Supplementary material for: Optimizing blood alcohol concentration measurement with breath alcohol meter and its preliminary application in evaluating CYP2E1 activity
Source: PLoS One. 2025 Oct 31;20(10):e0335312. doi: 10.1371/journal.pone.0335312 (PMC12578163; doi:10.1371/journal.pone.0335312)
Supplement: Attachment — Submitted filename: point by point response to each of the points.docx [file pone.0335312.s003.docx]

Subject: Revised Manuscript Submission: [PONE-D-25-32424] - [Optimizing Blood Alcohol Concentration Measurement with Breath Alcohol Meter and Its Preliminary Application in Evaluating CYP2E1 Activity]

Dear Dr. Jeong Hoon Pan

We sincerely appreciate your letter and the reviewers’ constructive comments on our manuscript entitled “[Optimizing Blood Alcohol Concentration Measurement with Breath Alcohol Meter and Its Preliminary Application in Evaluating CYP2E1 Activity]”. We are grateful for the opportunity to revise our manuscript and believe that it has been significantly improved as a result of the feedback provided.

We have carefully addressed all the comments from the reviewers and have incorporated the suggested changes into the manuscript. Below is a point-by-point response to the reviewers’ feedback. All corresponding revisions have been made in the main text and are highlighted for your convenience.

**Response to the Academic Editor’s Comments:**

Comment 1: The use of BrAC to evaluate CYP2E1 activity is insufficiently supported. Please provide mechanistic justification and, if possible, supporting data as a reviewer suggested to strengthen this claim.

The current title overstates the context. As actual optimization process was conducted, we recommend revising the title to better reflect the scope of the manuscript.

Response: Breath alcohol meter have been used by traffic police for many years to check drunk driving, but they cannot be directly applied to rats. We developed a methodology for estimating blood alcohol concentration in rats using a breath alcohol meter.

The first improvement involves the use of a meter equipped with an automatic suction pump, which reduces operational errors during sampling. The second optimization consists of setting the threshold for the blood alcohol concentration-time curve at 46 mg·dL⁻¹ instead of the previously used 20 mg·dL⁻¹. Studies have shown that alcohol metabolism below 20 mg·dL⁻¹ involves alcohol dehydrogenase, between 20–45 mg·dL⁻¹ it involves both alcohol dehydrogenase and CYP2E1, and above 46 mg·dL⁻¹ it is primarily mediated by CYP2E1. Therefore, blood alcohol concentration curves above 46 mg·dL⁻¹ better reflect the activity of CYP2E1.

The scientific rationale of this study is to optimize the methodology and explore the feasibility of using alcohol as a probe for preliminary assessment of CYP2E1 activity. Gas chromatography was used to measure blood alcohol concentration in rats to validate the accuracy of the breath alcohol meter. High-performance liquid chromatography was employed to monitor the metabolic process of chlorzoxazone, further verifying the feasibility of using alcohol as a probe to evaluate CYP2E1 activity. The experimental design is logically coherent and demonstrates clear innovation and scientific rigor. Additional arguments and references have been incorporated in the discussion section to strengthen the justification and enhance the alignment between the content and the title. The title of the manuscript is appropriate.

Comment 2: Please ensure that your manuscript meets PLOS ONE's style requirements, including those for file naming.

Response: We have carefully checked.

Comment 3: Please add clearer statements to the Methods section of your manuscript regarding the length of the experiment, the number of animals used, and the euthanization of animals at the conclusion of the experiment.

Response: We have carefully checked and supplemented the dosage of pentobarbital sodium used for animal euthanasia.

Comment 4: In your Methods section, please provide additional information regarding the experiments involving animals and ensure you have included details on (1) methods of sacrifice, (2) methods of anesthesia and/or analgesia, and (3) efforts to alleviate suffering.

Response: We confirm that the issues raised have already been addressed in the current version of our manuscript.

Comment 5: Statement on the role of funders.

Response: We have carefully checked. The funders had no role in study design, data collection and analysis, decision to publish, or preparation of the manuscript.

Comment 6: Your ethics statement should only appear in the Methods section of your manuscript.

Response: We have carefully checked.

Comment 7: In the online submission form, you indicated that the data presented in this study are uploaded during submission as a supplementary file and are openly available for readers upon request.

Response: We have carefully checked. The data presented in this study are uploaded during submission as a supplementary file and are openly available for readers upon request.

Comment 8: Please include captions for your Supporting Information files at the end of your manuscript, and update any in-text citations to match accordingly.

Response: We have revised the manuscript accordingly.

Comment 9: Please remove all personal information.

Response: We have revised the manuscript accordingly.

**Response to Reviewer #1’s Comments:**

Major Comment 1: The manuscript introduces a novel segmentation of the BrAC curve at 46 mg·dL⁻¹ but lacks a strong justification or citation to support this specific threshold.

Response: More than 90% of alcohol is metabolized in the liver into acetaldehyde, primarily through the actions of alcohol dehydrogenase and CYP2E1. Alcohol dehydrogenase has a relatively low Michaelis constant (Km) of less than 5 mM and becomes saturated once the blood alcohol concentration exceeds 15–20 mg·dL⁻¹. In contrast, CYP2E1 has a Km of approximately 10 mM, equivalent to about 46 mg·dL⁻¹, and is likely to play a dominant role in alcohol metabolism when blood alcohol concentration exceed this threshold^[12,15]^. This metabolic framework has been outlined in the Introduction section of our manuscript.

[12] Jiang Y, Zhang T, Kusumanchi P, Han S, Yang Z, Liangpunsakul S. Alcohol Metabolizing Enzymes, Microsomal Ethanol Oxidizing System, Cytochrome P450 2E1, Catalase, and Aldehyde Dehydrogenase in Alcohol-Associated Liver Disease. Biomedicines. 2020 Mar 4;8(3):50. doi: 10.3390/biomedicines8030050. PMID: 32143280; PMCID: PMC7148483.

[15] Zhang J, Cao Y, Jin Z, A R, Wang X, Zhang L, Hu Y, Xue Y. Dynamic CYP2E1 expression and metabolic activity changes in male rats during immune liver injury and sex differences in alcohol metabolism. PLoS One. 2025 Jun 18;20(6):e0325135. doi: 10.1371/journal.pone.0325135. PMID: 40531979; PMCID: PMC12176122.

Major Comment 2: The methods mention t-tests but do not address whether assumptions (e.g., normality, equal variance) were tested.

Response: We have revised the manuscript accordingly.

Major Comment 3: Figures 2-4 are referenced as key evidence, but no quantitative values (mean ± SD) are shown in the main text or figure captions.

Response: We have revised the manuscript accordingly.

Major Comment 4: The breathalyzer method is said to be "potentially field-deployable," but its translational application (e.g., human studies, clinical validation) is not discussed.

Response: We have supplemented in the Discussion section. Alcohol is a popular beverage often consumed during meals due to its stimulatory effects on the central nervous system and ability to enhance social interactions. Our computational approach, combined with breath alcohol monitoring, offers a non-invasive method to assess an individual's CYP2E1 metabolic activity. Furthermore, by analyzing the blood alcohol concentration-time curve, this strategy allows for estimation of the time required for complete alcohol clearance. Such an assessment is practically valuable for predicting when individuals can safely resume tasks requiring high precision, such as driving, after alcohol consumption. Additionally, this approach may serve as a biomarker for evaluating liver function.

Minor Comment 1: Use consistent units for BrAC (e.g., mg·dL⁻¹ vs. mg/100mL) throughout the manuscript.

Response: Response: We have revised the manuscript accordingly.

Minor Comment 2: Instead of referring to groups as "BCG" and "Control," consider more informative labels such as "Hepatitis model" vs. "Healthy control."

Response: After careful consideration, we have elected to retain the terms “BCG” and “CON” in this manuscript, as these designations have been consistently used across our previous publications ^[15,18,19]^. While we fully acknowledge the reviewer’s valid point regarding terminology clarity, we believe that maintaining this nomenclature ensures continuity with our earlier work. For clarity, we have explicitly indicated within the text that the “BCG group” refers to animals with BCG-induced hepatitis, and the “CON group” refers to normal control animals.

Minor Comment 3: Ensure all figure labels (e.g., A, B, C) are visible and explained clearly in the legends. Consider unifying the layout for all figures.

Response: We have carefully checked.

**Response to Reviewer #2’s Comments:**

Major comments: The title is over-stated since there is no “optimization” process for the breath alcohol meter’s performance. And even there is lack of reliable evidences for potential use of blood alcohol concentration as an indicator of hepatic CYP2E1 activity. Supporting data is required for this, for example, reactive oxygen species (ROS) level. For animal study, 56° (v/v) Erguotou Baijiu was used as an ethanol source, which is kind of distilled liquor, rather than use of pure ethanol.

Response:

1. Regarding the Optimization of Methods:

Breath alcohol meter have been used by traffic police for many years to check drunk driving, but they cannot be directly applied to rats. We developed a methodology for estimating blood alcohol concentration in rats using a breath alcohol meter.

The first improvement involves the use of a meter equipped with an automatic suction pump, which reduces operational errors during sampling. The second optimization consists of setting the threshold for the blood alcohol concentration-time curve at 46 mg·dL⁻¹ instead of the previously used 20 mg·dL⁻¹. Studies have shown that alcohol metabolism below 20 mg·dL⁻¹ involves alcohol dehydrogenase, between 20–45 mg·dL⁻¹ it involves both alcohol dehydrogenase and CYP2E1, and above 46 mg·dL⁻¹ it is primarily mediated by CYP2E1. Therefore, blood alcohol concentration curves above 46 mg·dL⁻¹ better reflect the activity of CYP2E1.

The scientific rationale of this study is to optimize the methodology and explore the feasibility of using alcohol as a probe for preliminary assessment of CYP2E1 activity. Gas chromatography was used to measure blood alcohol concentration in rats to validate the accuracy of the breath alcohol meter. High-performance liquid chromatography was employed to monitor the metabolic process of chlorzoxazone, further verifying the feasibility of using alcohol as a probe to evaluate CYP2E1 activity. The experimental design is logically coherent and demonstrates clear innovation and scientific rigor. Additional arguments and references have been incorporated in the discussion section to strengthen the justification and enhance the alignment between the content and the title. The title of the manuscript is appropriate.

2. Regarding the assessment of CYP2E1 metabolic activity:
   Currently, in the international research community, the assessment of CYP2E1 activity is primarily based on monitoring the blood concentration of chlorzoxazone rather than reactive oxygen species (ROS) levels. This is because ROS in vivo can be generated through multiple pathways, making it difficult to distinguish which portion is specifically produced by CYP2E1 metabolism. Therefore, the blood concentration-time curve of chlorzoxazone remains the gold standard for evaluating the feasibility of using high-concentration alcohol as a probe for CYP2E1 activity.
3. Regarding the use of 56° (v/v) Erguotou Baijiu in animal experiments:
   Using 56% distilled liquor in animal experiments better approximates the biological realism of human alcohol consumption. While pure ethanol represents an “idealized single-component” substance, distilled spirits commonly consumed by humans (such as Erguotou) contain not only ethanol but also trace volatile compounds (e.g., esters, acids, and aldehydes, typically accounting for <1%). It is this “interaction among multiple constituents” that truly reflects the biological response in humans after alcohol intake. The use of pure ethanol would overlook the potential effects of these “non-ethanol components,” leading to a disconnect between experimental outcomes and the actual physiological processes of human drinking, thereby reducing the predictive validity of the model.

In our studies—including breath alcohol meter, gas chromatography and liquid chromatography experiments—we consistently used distilled liquor to ensure comparability. This approach does not compromise the comparison of CYP2E1 metabolic activity. Even if trace compounds exert effects, such influence would be consistent across all three methods, allowing for homogeneous comparison. Considering the enhanced biological relevance to human alcohol consumption, the benefits outweigh the drawbacks.

4. Regarding the BCG-induced hepatitis rats:
   In previous studies, we have repeatedly employed the BCG-induced hepatitis rats ^[15,18,19]^ and observed downregulation in the activity of multiple enzymes, including CYP2E1. CYP2E1 activity can serve as a biomarker reflecting liver function. Relevant discussions and potential applications have been added to the Discussion section of the manuscript.

Minor Comments:

Use of abbreviation; several abbreviations were not defined in the main text. Such as BCG, LPS.

2.1. Materials: some materials and equipment are just listed up, not in complete sentence.

Figure legends: insufficient information and description for the figures. And typing errors, for example, p value *P <0.05, *P <0.01.

Inconsistency in use of terms. For example, 56°Alcohol, 56% ethanol

Response: We have revised the manuscript accordingly.

We have also thoroughly proofread the manuscript to improve clarity and language fluency. We believe that all the reviewers’ concerns have been adequately addressed in the revised version and that the manuscript is now much stronger.

Thank you again for considering our work. We look forward to your positive response.

Sincerely,

[Yongzhi Xue]

[Baotou Medical College]

[xyzhxyzh68@sohu.com (YZX)]

[2025.9.9]
