# Supplementary material for: Optimizing blood alcohol concentration measurement with breath alcohol meter and its preliminary application in evaluating CYP2E1 activity
Source: PLoS One. 2025 Oct 31;20(10):e0335312. doi: 10.1371/journal.pone.0335312 (PMC12578163; doi:10.1371/journal.pone.0335312)
Supplement: Attachment — Submitted filename: point_by_point_response_to_each_of_the_points_auresp_2.docx [file pone.0335312.s004.docx]

Subject: Revised Manuscript Submission: [PONE-D-25-32424] - [Optimizing Blood Alcohol Concentration Measurement with Breath Alcohol Meter and Its Preliminary Application in Evaluating CYP2E1 Activity]

Dear Dr. Jeong Hoon Pan

We are grateful for the opportunity to revise our manuscript and believe that it has been significantly improved as a result of the feedback provided.

We have carefully addressed all the comments from the reviewers and have incorporated the suggested changes into the manuscript. Below is a point-by-point response to the reviewers’ feedback. All corresponding revisions have been made in the main text and are highlighted for your convenience.

**Response to Reviewer #2’s Comments:**

Major Comment 1: Authors should address limitations of alcohol meter for CYP2E1 activity monitoring.

Response: In the Discussion section of our revised manuscript, we have addressed the limitations associated with the use of breath alcohol meter. It is noteworthy that breath alcohol testing requires a sufficient alcohol dose, as CYP2E1-mediated metabolism becomes dominant only when the blood alcohol concentration exceeds 46 mg·dL⁻¹. Essentially, breath alcohol meter serve as tools for detecting alcohol concentration rather than directly measuring enzyme activity. This inherent limitation of indirect detection is similarly observed in the conventional chlorzoxazone-based method. To address this, procedural optimizations can be implemented to minimize indirect errors, while techniques such as quantifying CYP2E1 protein and mRNA expression can provide complementary evidence. Ultimately, this approach facilitates a "rapid screening (breath alcohol testing) + precise verification (direct techniques)" framework, which not only enables on-site identification of impaired alcohol metabolism but also clarifies the role of CYP2E1 activity in the process. Despite the limitations noted above, the methodology established in this study offers a novel and non-invasive approach for the dynamic monitoring of CYP2E1 metabolic activity, thereby opening new avenues for further research and application.

Minor Comment 1：Method 2.3, there is an error in the order of collection time points: 20, 40, 60, 120, 240, 180, 300, 420, 540 min.

Response: We have revised the manuscript accordingly.

Thank you again for considering our work. We look forward to your positive response.

Sincerely,
[Yongzhi Xue]
[Baotou Medical College]
[xyzhxyzh68@sohu.com (YZX)]
[2025.9.30]
